# Rethinking Animal Welfare in a Globalised World: Cultural Perspectives, Challenges, and Future Directions

**DOI:** 10.3390/ani15060891

**Published:** 2025-03-20

**Authors:** Sarah Oxley Heaney, Michelle Szydlowski, Kristine Hill, Jes Hooper

**Affiliations:** 1 Department of Social and Political Sciences, Philosophy, and Anthropology (SPSPA), Faculty of Humanities, Arts and Social Sciences, University of Exeter, Devon EX4 4QJ, UK; 2 Department of Biology, College of Arts and Science, Miami University, Oxford, OH 45056, USA; 3 Department of Philosophy and Social Sciences, Philosophical Faculty, University of Hradec Králové, 500 02 Hradec Králové, Czech Republic

## 1. Introduction

In an increasingly interconnected world, human and other-than-human-animal welfare has emerged as a significant global concern. The welfare of these entangled lives, whether through anthropocentric, biocentric, or other-than-human-centred posthuman lenses, face challenges arising from cultural, political, and social boundaries. Urbanisation, globalisation, pandemics, and evolving ethical standards have amplified the focus on how other-than-human animals are treated across various settings. Realising that both human and other-than-human-animal societies co-evolve and interrelate, often beyond human imaginations, human societies must navigate the complexities of their relationships with other-than-human animals in the era now classified as the Anthropocene. The need for a nuanced understanding of the interconnectedness of human and other-than-human-animal welfare is becoming increasingly urgent. By “animal welfare”, we refer to the welfare of *all* animals, including humans, unless specified otherwise. This acknowledges the trans-species nature of our intertwined lives in the biosphere.

## 2. Animal Welfare

Animal welfare is often described as the physical, mental, and emotional wellbeing of other-than-human animals. Humans arguably have a responsibility to ensure the welfare needs of domesticated and/or otherwise-captive animals are met, and some argue that those of wild-living other-than-human animals should also be considered (e.g., [1,2]). However, beliefs about what the needs of a species, or an individual, entail and what constitutes good practice can vary greatly. An understanding of different cultural perspectives is especially pertinent for researchers and animal advocates concerned with the welfare of animals situated in touristic, foreign (relative to the researcher), or culturally diverse contexts.

For this Special Issue, we welcomed manuscripts that addressed animal welfare from a cross-cultural perspective, including those considering more-than-human cultures. We were particularly interested in submissions offering theoretical contributions towards more ethical and symbiotic relations between humans and other-than-human animals. Finally, we encouraged submissions addressing the decolonization of animal welfare in academia and/or practice.

The articles in this Special Issue shed light on diverse issues surrounding other-than-human-animal welfare, from the management of community cats in urban China to the ethical dilemmas of elephant tourism in Nepal, and the controversial civet coffee production industry in Asia. Each study provides a unique perspective on how cultural contexts, governance, and human–other-than-human relationships shape our understanding of animal welfare. Despite the diversity of these issues, there is a unifying call for more humane treatment of species and individuals, necessitating cross-cultural understanding, tailored policies, and innovative solutions.

Animal welfare encompasses a broad range of concerns, often addressing the well-being of various species in human-controlled environments e.g., [3,4]. In this Special Issue, for example, Kamal and colleagues’ paper *Recognising Zooeyia to Promote Companion Animal Welfare in Urban Bangladesh* and Gu and colleagues’ article titled *A Survey of Public Opinion on Community Cats’ General Health and Relationship Quality with Residents in Urban China* highlight the pressing need to improve the welfare of other-than-human animals living closely alongside humans [5,6]. These studies emphasise that animal welfare is not solely an ethical issue but is intrinsically linked to the health, safety, and community well-being of individuals of all species. Furthermore, the examination of animal welfare in different cultural contexts, as seen in *Mandan, Hidatsa, and Arikara Nation Perspectives on Rez Dogs on the Fort Berthold Reservation in North Dakota, U.S.A.* by Cardona and colleagues and *Ubiquitous Love or Not? Animal Welfare and Animal-Informed Consent in Giant Panda Tourism* by Fennell and Guo, underscores the diverse factors influencing human–other-than-human-animal relationships and the growing recognition of many species as sentient beings deserving of care and respect [7,8].

The aforementioned articles of Cordonal et al. and Gu et al. are supported by Rogers et al. in *Perceptions of Cross-Cultural Challenges and Successful Approaches in Facilitating the Improvement of Equine Welfare* [7,8,9]. Community engagement and collaboration are essential components for improving animal welfare. Cordona et al. emphasise the need for these interventions to be culturally relevant if they hope to address the welfare of reservation dogs [9]. Accentuating the importance of local partnerships and understanding cultural contexts is vital to achieving successful outcomes. Rogers et al. advocate for working alongside local communities to foster trust and collaboration as they discuss the challenges of managing animal welfare across different countries and cultural frameworks [9]. Similarly, Cui and colleagues’ work, *Farm Animal Welfare Is a Field of Interest in China: A Bibliometric Analysis Based on CiteSpace,* confirms a growing interest in farm animal welfare in China, and the need for increased collaboration amongst authors and institutions within China and less anthropocentric-focused policy efforts for more effective outcomes [10].

Cultural beliefs and practices play a pivotal role in shaping attitudes toward animal welfare. For instance, the study by Kamal et al. explores the stigma surrounding companion animal ownership in Bangladesh, illustrating how cultural perceptions can impede welfare efforts. Moreover, the nuanced understanding of animal welfare can vary significantly among populations, as demonstrated in *Perceptions of Farm Animal Sentience and Suffering: Evidence from the BRIC Countries and the United States* by Mata et al. [11] This study reveals that attitudes toward animal sentience differ not only between countries but also within them, accentuating the subtle need for intra-country, culturally sensitive approaches to animal welfare. Some articles peel off layers of complexities within human–other-than-human-animal relationships, showing how they are influenced by historical, cultural, and social factors within communities themselves. For example, Kamal et al. demonstrate how caretakers of companion animals in Bangladesh report significant social and psychological benefits, despite the societal stigma associated with their ownership [5]. Cordonal and colleagues’ “rez dog” study highlights how Indigenous communities maintain deep cultural ties with free-roaming dogs, reflecting their unique perspectives on animal welfare, which are often misunderstood and clash with or are neglected by those who govern, and attempts to impose animal welfare solutions [7].

Effective governance and policy frameworks when renegotiating a one-size-fits-all approach are shown as being crucial for advancing animal welfare. *Beyond the Unitary State: Multi-Level Governance, Politics, and Cross-Cultural Perspectives on Animal Welfare* by Chaney et al. discusses how political contexts and civil society can influence the development of animal welfare policies in the UK, highlighting the importance of multi-level governance in addressing animal welfare concerns [12]. The challenges associated with implementing policies are evident in Bangladesh, which underscores the gaps in enforcing the Animal Welfare Act (2019) and the implications for companion animal welfare. Meanwhile, Cui et al. stress the Chinese institutional power dynamics within the agricultural machine—which prioritises economic aspects and certain “livestock” species—that prevent community voices from being heard [10]. These aspects compel contributors to consider the ethical and moral dimensions of animal welfare. Ethical dilemmas surrounding the use of other-than-human animals in various contexts are a recurring theme. For instance, Fennel and colleagues’ *Ubiquitous Love or Not? Animal Welfare and Animal-Informed Consent in Giant Panda Tourism* and Szydlowski’s article *Wicked Problems, Novel Solutions: Nepalese Elephant Tourism and Conservation* on Nepalese elephants both raise critical questions about animal-informed consent, challenging readers to consider whether giant pandas and elephants genuinely “agree” to their roles as tourist attractions [8,13].

Several authors discuss how economic factors are intricately linked to animal welfare. Cui and colleagues’ study *Farm Animal Welfare Is a Field of Interest in China: A Bibliometric Analysis Based on CiteSpace* demonstrates how addressing welfare concerns can lead to economic benefits such as improved growth and product quality [10]. The dual focus on welfare and economic viability is also evident in *Wicked Problems, Novel Solutions: Nepalese Elephant Tourism and Conservation* and *Thinking with Civets: The Role of Zoos in the Decolonisation of Animal Tourism,* both of which discuss the complex relationship between economic reliance on animal tourism and the ethical treatment of animals [13,14]. *Perceptions of Cross-Cultural Challenges and Successful Approaches in Facilitating the Improvement of Equine Welfare* and *Mandan, Hidatsa, and Arikara Nation Perspectives on Rez Dogs on the Fort Berthold Reservation in North Dakota, U.S.A*. also identify challenges such as limited financial resources [7,9].

The commodification of animals in tourism is further explored in *Thinking with Civets: The Role of Zoos in the Decolonisation of Animal Tourism,* which examines how colonial legacies continue to impact animal welfare in tourism contexts [14]. Both Cardona et al.’s article on “rez dog” and Hooper’s paper on civets offer a critical examination of colonial legacies and explore how decolonisation could affect the practices of care and commodification analysed in their research [7,14]. *Thinking with Civets* reveals ongoing impacts on the commodification of animals and illustrates how the history of colonialism continues to shape contemporary animal tourism practices [14].

Culture itself is being increasingly recognised as existing in various forms across species (e.g., [15,16,17]). Acknowledging the nuances unveiled by considering cultural differences and decolonisation for *all* animals is paramount in unpacking human–other-than-human-animal relationships. These considerations are foregrounded in Szydlowski’s article, where she reflects upon the effects on *all individuals* wrapped up in animal welfare concerns. Here, Szydlowski illustrates the need to rethink current ideologies surrounding the use of other-than-human animals in the promotion of both conservation and tourism practices. Elephants serve as a nucleus for ongoing political, land use, and environmental justice debates throughout SE Asia. In range countries, such as Nepal, elephants and their caregivers face additional challenges thanks to their liminal status as tourist transport, objects of worship, subjects of conservation practice, and co-workers in forestry efforts aimed at endangered-species conservation. Arguing that finding “common ground” requires the use of novel problem-solving techniques, Szydlowski suggests that only when community members, international interests *and* endangered individuals themselves are valued equally will positive change occur. This emphasis brings attention to the bespoke welfare needs of persons, human or other-than-human, rather than creating universal, coarse remedies intended to be panaceatic but which often result in discordant outcomes.

## 3. Conclusions

As we confront the multifaceted challenges of interrelated animal welfare in a globalised world, it becomes clear that understanding animal welfare through culturally sensitive, ethically grounded, and historically informed lenses is paramount. While the included articles discuss the broader implications of animal welfare in the Global South, we recognise the growing need for more ethical practices that respect both cultural contexts and animal rights in both hemispheres, while being cognisant of the stratified, historically layered, and fluid boundaries that form our world.
